# Pseudopancreatic Cyst Extending into the Mediastinum in a 7-Year-Old Child

**DOI:** 10.1055/s-0039-1688802

**Published:** 2019-06-17

**Authors:** Mostafa Kotb, Ahmed Oshiba, Khaled Ashour

**Affiliations:** 1 Department of Pediatric Surgery, Alexandria University Faculty of Medicine, Alexandria, Egypt

**Keywords:** pancreatic pseudocyst, mediastinal extension, cystogastrostomy

## Abstract

Mediastinal pseudopancreatic cyst (MPP) is the extension of a pancreatic pseudocyst through esophageal or aortic hiatus into the posterior mediastinum. It can produce a range of manifestations caused by compression by the cyst, for instance, odynophagia, dysphagia, pericardial, or pleural effusion. Here we report a case of MPP in a 7-year-old child who was presented with repeated chest infections and left pleural effusion. It was successfully drained by cystogastrostomy.

## Introduction

Pancreatic pseudocyst (PP) is a localized collection of pancreatic secretions, rich in amylase and other enzymes that is lined by fibrous tissue and do not have an epithelial lining. Only few cases had been reported in childhood in literature. Mediastinal extension of a PP is even rarer. Depending on the mass effect it exerts, and it can cause pericardial or pleural effusion, dysphagia, or odynophagia. 1 We present a case of mediastinal pseudopancreatic cyst (MPP) in a 7-year-oldchild who present to our department without a prior history of acute pancreatitis or significant trauma. After clinical examination and imaging, it turned out to be a pseudopancreatic cyst extending to the mediastinum through the diaphragm. The patient underwent cystogastrostomy with uneventful postoperative course.

## Case Report

**Fig. 1:**
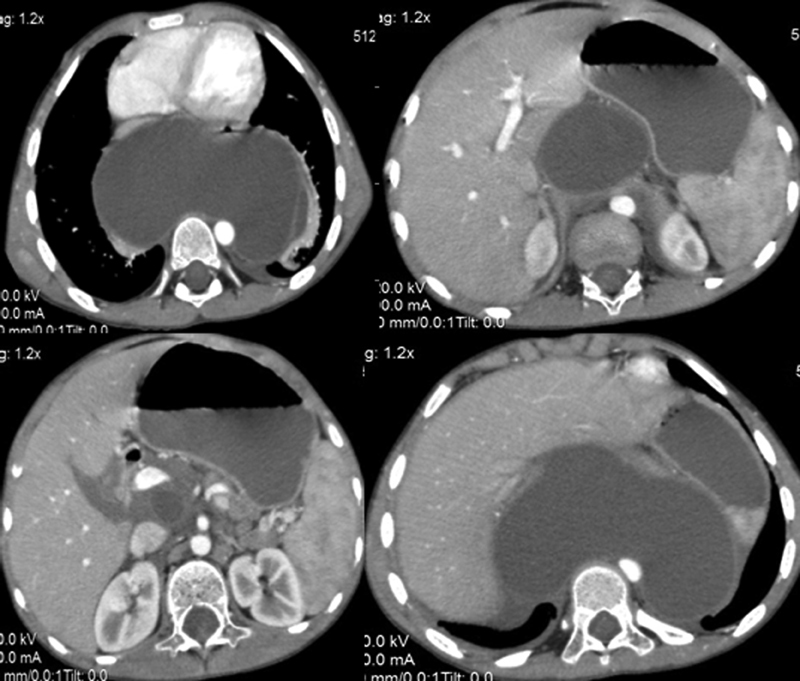
CT abdomen showing fluid containing cystic mass behind the stomach around the pancreas extending up to the chest. CT, computed tomography.

A 7-year-old male child was presented to the Pediatric Department in Shatbi University Hospital with recurrent chest infections since 1 year. Repeated chest X-ray showed left pleural effusion. The child was managed conservatively and discharged from the pediatric department. During the course of follow-up for the respiratory condition an abdominal ultrasound was ordered to investigate a new onset minor abdominal discomfort. An abdominal cyst was found which, otherwise, was not clinically palpable during abdominal examination. Subsequent computed tomography (CT) scanning of the abdomen and pelvis with intravenous contrast revealed a retroperitoneal thick-walled fluid filled mass, measuring approximately 11.5 × 13 cm in close relation to the main pancreatic duct with inflammation of the adjacent pancreatic tissue. Provisional reports indicated a pseudo-pancreatic cyst with further extension into the posterior mediastinum through one of the diaphragmatic hiatus ( Fig. 1 ). Serum amylase and lipase were markedly elevated (amylase: 45,630 U/I and lipase: 180,000 U/I).

The decision after surgical consultation was to perform laparotomy. The child was prepared for the operation. Through an upper midline incision, the abdomen was explored. This revealed a large, smooth, and fluctuant mass behind the stomach extending up to the posterior mediastinum through the esophageal hiatus, mostly arising from the pancreas. Aspiration of the coffee ground contents was done followed by a drainage procedure by anastomosing the posterior wall of the stomach to the anterior wall of the cyst wall (cystogastrostomy) using running 4/0 vicryl sutures. A Penrose drain was inserted in the left upper quadrant followed by a layered closure. The postoperative course was uneventful. Nasogastric suction and intravenous fluids were continued for 5 days, after which oral feedings were gradually given. The drain was removed after 5 days and the child was discharged from the hospital on the 9th postoperative day. Follow-up abdominal ultrasound was done after 2 weeks and revealed dramatic improvement regarding the size of the cyst. Serum amylase gradually fell to the normal limits. A repeat ultrasound confirmed complete resolution of the cyst after 6 months. The patient is doing well after 1 year of regular follow-up visits.

## Discussion

PP is a major and common complication of pancreatitis, though few cases are reported in children. Approximately 80% of PPs are located within the head and the body of the pancreas, while the rest lie extrapancreatic in areas, such as mediastinum, liver, spleen, pelvis, and neck. 2 Since the first description of a case of MPP in an adult patient, only few cases had been reported in children. To our best knowledge, only eight cases had been reported in addition to ours ( Table 1 ). It is postulated that during the acute phase of pseudocyst formation, the fluid may track along the path of least resistance to gain access into the mediastinum via aortic or esophageal hiatus of the diaphragm hiatus. Later, the outer wall gets organized to form a pseudocyst. 3 The fluids mostly travel through the esophageal and aortic hiatus into the posterior mediastinum 4 However, If the extension was through the vena caval hiatus or the foramen of Morgagni, MPP will be located in the middle and anterior mediastinum, respectively. 5

**Table 1:** Cases of MPP in children reported in literature

Study	Age	Etiology	Main complaint	History of pancreatitis	Abdominal mass	Route of extension	Management
Laird and Clagett 6	15 y	Post traumatic	Anorexia, nausea, vomiting	No	No	Esophageal hiatus	Puestow procedure
Galligan and Williams 7	10 y	Idiopathic	Anorexia, nausea, vomiting	No	Yes	Esophageal hiatus	Cystogastrostomy
Kirchner et al 8	7 mo	Idiopathic	Dyspnea	No	No	Foramen of Morgagni	Roux-en-Y cysto-jejunostomy
Sharma et al 9	8 y	Post traumatic	Chest pain, dyspnea	No	No	Traumatic diaphragmatic hernia	Cysto-gastrostomy
Crombleholme et al 10	2 y	Ductal anomaly	Vomiting	No	No	Esophageal hiatus	Roux-en-Y cysto-jejunostomy
	7 y	Ductal anomaly	Abdominal pain	Yes	No	Esophageal hiatus	Puestow's procedure
Bonnard et al 11	11 y	Ductal anomaly	–	–	–	Not given	Thoracoscopic drainage
Nabi et al 12	11 y	Idiopathic	Abdominal pain	Yes	No	Not given	Transgastric endoscopic drainage
Current case	7 y	Idiopathic	Dyspnea	No	No	Esophageal hiatus	Cystogastrostomy

As MPP decompresses through the diaphragm, it is usually not felt while performing abdominal examination. It can present with nonspecific manifestations caused by compression by the cyst. This includes abdominal pain, gastrointestinal upsets, chest pain, dyspnea, dysphagia, odynophagia, and symptoms of pericardial or pleural effusion like our case. 13

Owing to the vague nature of symptoms, imaging remains the cornerstone of diagnosis of such a rare disease. While chest radiography is not diagnostic but can illustrate lower mediastinal widening, retro or paracardiac well-defined opacity in addition to associated features, such as pleural or pericardial effusion. 14 Although ultrasound is accurate in diagnosing PP, it cannot detect the existing mediastinal extension. 15 Contrast-enhanced CT is a valuable modality not just in identifying the pseudocyst but also establishing its relationship with surrounding structures. It appears as a thin-walled low attenuation peripherally enhancing cyst. 16 MRCP (magnetic resonance cholangiopancreatography) is as effective as ERCP (endoscopic retrograde cholangiopancreatography) to demonstrate the track between the abdominal cyst and the mediastinal cyst, as well as ductal morphology. 17

The approach to treatment of MPP is individualized depending on the underlying etiology, ductal anomaly, and whether the patient is symptomatic or not. Indications for intervention include increasing pseudocyst size or persistence over 4 to 6 weeks. 18 Surgical treatment has often been used for therapeutic management of patients with mediastinal pseudocyst and these can vary from pancreatic resections to external or internal drainage, targeting the abdominal component to reduce the pressure that causes patency of any communicating tract. 19 In children, three cases underwent cystogastrostomy, roux-en-Y cystojejunostomy was done in two cases and similarly the Puestow's procedure. Each of the endoscopic ultrasound (EUS) assisted endoscopic drainage through transgastric approach and thoracoscopic drainage were done in one case and claimed by the authors to obviate the need for cystenterostomy. 11 12

## Conclusion

Although extremely rare, the diagnosis of MPP should be considered in the differential diagnosis of any cystic mediastinal mass. The finding of a thin-walled cystic mass in the posterior or middle mediastinum in continuity with pancreas in addition to an elevated serum amylase level can establish the definitive diagnosis.

## Notes

New Insights and the Importance for the Pediatric Surgeon Pseudopancreatic cyst is uncommon in children and mediastinal extenstion is extremely rare with few cases reported in literature. It represents a diagnostic challenge and should be included in the differential diagnosis of cystic mediastinal swellings.
